# Colorimetric detection of the potent carcinogen aflatoxin B_1_ based on the aggregation of L-lysine-functionalized gold nanoparticles in the presence of copper ions

**DOI:** 10.3389/fnut.2024.1425638

**Published:** 2024-06-06

**Authors:** Kaori Sánchez-Carrillo, David Quintanar-Guerrero, Miguel José-Yacamán, Abraham Méndez-Albores, Alma Vázquez-Durán

**Affiliations:** ^1^Unidad de Investigación Multidisciplinaria L14 (Alimentos, Micotoxinas, y Micotoxicosis), Facultad de Estudios Superiores Cuautitlán (FESC), Universidad Nacional Autónoma de México (UNAM), Cuautitlán Izcalli, Mexico; ^2^Laboratorio de Posgrado en Tecnología Farmacéutica, FESC, UNAM, Cuautitlán Izcalli, Mexico; ^3^Applied Physics and Materials Science Department and Center for Materials Interfaces in Research and Applications (¡MIRA!), Northern Arizona University, Flagstaff, AZ, United States; ^4^Laboratorio de Fisicoquímica L414, FESC, UNAM, Cuautitlán Izcalli, Mexico

**Keywords:** gold nanoparticles, aflatoxin B_1_, colorimetric detection, aggregation, maize

## Abstract

L-lysine functionalized gold nanoparticles (AuNPs-Lys) have been widely used for the detection of worldwide interest analytes. In this work, a colorimetric assay for the detection of the carcinogen aflatoxin B_1_ (AFB_1_) based on the aggregation of AuNPs-Lys in the presence of copper ions was developed. For this purpose, AuNPs were synthesized in citrate aqueous solution, functionalized, and further characterized by UV–Vis, fluorescence, Fourier transform infrared spectroscopy (FTIR), nanoparticle tracking analysis (NTA), dynamic light scattering (DLS), and transmission electron microscopy (TEM). In general, AuNPS-Lys (~2.73 × 10^11^ particles) offered a clear colorimetric response in the presence of AFB_1_ and Cu^2+^ ions showing linearity in the range of 6.25 to 200 ng AFB_1_/mL, with a detection limit of 4.18 ng AFB_1_/mL via photometric inspection. Moreover, the performance of the proposed methodology was tested using the 991.31 AOAC official procedure based on monoclonal antibodies in maize samples artificially contaminated with AFB_1_. There was a good agreement between the measured AFB_1_ concentrations in both assays, the average recoveries for the colorimetric and immunoaffinity assays were between 91.2–98.4% and 96.0–99.2%, respectively. These results indicated that the colorimetric assay could be used as a rapid, eco-friendly, and cost-effective platform for the quantification of AFB_1_ in maize-based products.

## 1 Introduction

Mycotoxins—fungal secondary metabolites with toxic properties—represent significant challenges to food safety and public health worldwide. Therefore, mitigation strategies involving prevention, monitoring, and control are fundamental to minimize the risks and ensure the safety of food and feed supplies. Aflatoxins, a subgroup of mycotoxins, are mainly produced by fungi of the genus *Aspergillus*, particularly *A. flavus*, *A. parasiticus*, and *A. nomius* (1). Four major toxins are produced including aflatoxin B_1_ (AFB_1_), aflatoxin B_2_ (AFB_2_), aflatoxin G_1_ (AFG_1_), and aflatoxin G_2_ (AFG_2_). From this group, AFB_1_ is by far the most potent hepatotoxic, carcinogenic, teratogenic, and mutagenic compound of natural origin. For this reason, the International Agency for Research on Cancer classified AFB_1_ as a human Group 1 carcinogen (2).

Mexican regulations established the maximum levels of total aflatoxins (the sum of B- and G-series) allowed in maize intended for human consumption, action levels are set at 20 ng/g (NOM-188-SSA1-2002) (3). To meet this objective, the quantification of aflatoxins has been performed by different methodologies such as thin layer chromatography (TLC), high-performance or ultra-performance liquid chromatography (HPLC or UPLC), enzyme-linked immunosorbent assays (ELISA), liquid chromatography coupled to mass spectrometry (LC-MS), fluorescence polarization immunoassays (FPIA), and polymerase chain reaction (PCR), among others. These techniques vary in sensitivity, selectivity, and complexity; however, the drawbacks of these methodologies are that they are unsuitable for rapid and real-time applications as they require expensive instruments, are time-consuming, and often demand skilled personnel. Therefore, rapid, and robust assays such as colorimetric platforms have emerged in recent years for the rapid and easy detection of aflatoxins (4).

Since the development of materials science and technology, different materials such as nanoparticles, quantum dots, nanotubes, nanowires, and two-dimensional nanostructures have been proposed for the fabrication of diverse sensor platforms (5). The ability of these materials to accelerate the signal transduction process results in the development of rapid, sensitive, cost-effective, and highly specific sensors. Recently, gold nanoparticles (AuNPs)-based colorimetric sensors have received great interest due to their distinctive properties such as facile synthesis methods, good stability, shape and size control, optical properties, and straightforward surface functionalization. In general, colorimetric platforms are based on the localized surface plasmon resonance (SPR) of AuNPs, as well as on the aggregation process resulting from the interaction between the analyte and the functionalized AuNPs. This aggregation phenomenon can also be followed by a naked-eye color change due to the electromagnetic coupling among the SPR of nearby AuNPs (6). In the literature, various studies reported the analysis of AFB_1_ using chromogenic reactions based on noble metal nanoparticles (7–10). However, colorimetric methodologies using the aggregation or anti-aggregation approach for the detection of AFB_1_ are still meager. For instance, Du et al. (11) utilized mercury ions to induce the de-aggregation of AuNPs functionalized with L-lysine (AuNPs-Lys). The visual detection limit of the proposed methodology was 15 ng/g with a quantitative detection limit of 1.1 ng/g. However, the toxicity of mercury to humans and the environment compromises its application in a real-world scenario since mercury cannot be degraded into non-toxic compounds. Thus, the development of eco-friendly colorimetric platforms for the detection of AFB_1_ and other mycotoxins is still needed. Until today, there is currently a lack of information regarding the use of copper ions for the detection of AFB_1_ based on the aggregation of AuNPs-Lys. Consequently, this research was conducted to synthesize, characterize, evaluate, and compare the potential of the AuNPs-Lys system for the colorimetric detection of AFB_1_ in aqueous media and in maize samples artificially contaminated with AFB_1_.

## 2 Materials and methods

### 2.1 Materials

Tetrachloroauric(III) acid trihydrate (CAS No. 16961-25-4), sodium citrate tribasic dihydrate (CAS No. 6132-04-3), L-lysine (CAS No. 56-87-1), copper (II) chloride (CAS No. 7447-39-4), hydrochloric acid 37% (CAS No. 7647-01-0), nitric acid 70% (CAS No. 7697-37-2), AFB_1_ from *Aspergillus flavus* (CAS No. 1162-65-8), dimethyl sulfoxide (CAS No. 67-68-5), methanol/HPLC grade methanol (CAS No. 67-56-1), and sodium chloride (CAS No. 7647-14-5) were purchased from Merck KGaA, Darmstadt, Germany. Milli-Q water (resistivity 18.25 MΩ·cm at 298 K and a TOC value ≤5 μg/L) was utilized throughout and was obtained via a Milli-Q purification system (Millipore Co., Bedford, MA, United States). Before the synthesis protocol, all glassware was exhaustively cleaned with a mixture of hydrochloric acid and nitric acid (3:1 v/v) to remove any potential contaminant.

### 2.2 Gold nanoparticles (synthesis and L-lysine functionalization)

Gold nanospheres were synthesized by the Turkevich methodology (12). Briefly, 40 mL of 0.5 mM freshly prepared gold (III) chloride trihydrate were heated to its boiling point with constant stirring; subsequently, 10 mL of preheated sodium citrate tribasic dihydrate (50 mM) were added. The mixture was left with vigorous stirring at 70°C for 30 min to allow the reduction of the gold ions and the subsequent nanoparticle formation. Thereafter, gold nanoparticles (AuNPs) were washed (centrifugation and redispersion) three times (7,000 × g, 3 min cycles) and resuspended in Milli-Q water for further functionalization. To find the optimal AuNPs:Lys molar ratio for the functionalization of gold surfaces, eight different concentrations of L-lysine (0, 0.2, 0.4, 0.6, 0.8, 1.0, 2.0, and 3.0 mM) were added to a set of flasks containing 3 mL of the as-prepared AuNPs (0.3 mM) under stirring for 30 min at room temperature. Thus, adequately L-lysine functionalized AuNPs (AuNPs-Lys)—stable against agglomeration—were attained for all subsequent trials. Representative images were acquired with a single-lens-reflex camera α58 (SONY Corp. Bangkok, Thailand).

### 2.3 Aggregation experiment of L-lysine functionalized nanoparticles (AuNPs-Lys) via Cu^2+^ ions

Since Cu^2+^ ions have the advantage to induce the agglomeration of the AuNPs-Lys system, different concentrations of the metal ion were evaluated to find the optimal concentration to trigger nanoparticle aggregation. For such, copper (II) chloride at concentrations ranging from 7.8 to 1,000 μM were added to the AuNPs-Lys system (0.3 mM) and vortexed for 10 s leaving the reagents interacting at room temperature for 5 min. Afterward, representative aliquots were taken to evaluate AuNPs-Lys stability through UV–Vis spectroscopy. The appearance of a broad absorption band and a red shift in the absorption maximum were indicative of the AuNPs-Lys aggregation.

### 2.4 Characterization

#### 2.4.1 UV–Vis and fluorescence spectroscopies

Spectral analysis was done using a Cary 8454 UV–Vis Diode Array System spectrophotometer (Agilent Technologies, Santa Clara, CA, United States). Five spectra were collected in the range of 400–800 nm at room temperature in a 1 cm path quartz cell, and the characteristic SPR centered at 522 nm, which is typical for AuNPs, was registered. Milli-Q water served as a blank. The fluorescence spectra of the aqueous suspensions of AuNPs were measured using a LS-55 fluorescence spectrophotometer (Perkin Elmer, Waltham, MA, United States). Spectra were recorded in the wavelength range of 300–600 nm at an excitation wavelength of 325 nm using equally wide excitation and emission slits (10 nm).

#### 2.4.2 FTIR spectroscopy

The FTIR spectra of AuNPs were acquired in a Frontier SP8000 NIR/MIR spectrophotometer (Perkin Elmer, Waltham, MA, United States) accessorized with an in-compartment diamond ATR additament (DuraSamplIR II, Smiths Detection, Warrington, United Kingdom). Briefly, undiluted samples (250 μL) of AuNPs were placed on the ATR crystal and measured in transmittance mode. The spectra were recorded from 4,000 to 400 cm^−1^ at a resolution of 4 cm^−1^. A background spectrum was obtained against air, and the spectra were collected in quintuplicate (the average value was reported). The main vibrational bands were analyzed using the Spectrum 10.4.2 software.

#### 2.4.3 Nanoparticle tracking analysis

A NanoSight NS300 (Malvern Instruments, Worcestershire, United Kingdom) equipped with a 532 nm green laser module was utilized. Data were recorded using a 20× objective and a 60 s video clip. Three 60 s video measurements were recorded to provide the average mean and mode values as well as the concentration of AuNPs. Sample preparation was as follows: 10 μL of AuNPs were diluted with Milli-Q water to a total volume of 10 mL. Thereafter, an aliquot was taken with a sterile syringe and injected into the equipment’s chamber, preventing the formation of air bubbles. The dilution of the sample was to ensure the presence of 10^6^–10^9^ nanoparticles per milliliter. The capture setting parameters used were camera type: super-high-sensitivity complementary metal oxide semiconductor camera; camera level: 9; slider shutter: 607; slider gain: 15; number of frames: 1,498; temperature: 21.1°C; and viscosity: (water) 1.0 cP. The software used to capture and analyze data was nanoparticle tracking analysis (NTA) 2.0 Build 127.

#### 2.4.4 Particle size, polydispersity index, and zeta-potential measurements

A particle size analyzer (ZetaSizer Pro, Malvern Instruments, Worcestershire, United Kingdom) capable of both particle size (using dynamic light scattering) and zeta-potential measurements (using Doppler electrophoresis) was used. An aliquot of 500 μL of AuNPs diluted in 5 mL of Milli-Q water was prepared, and the sample was slowly passed through a disposable capillary cell DTS1070 to avoid the formation of air bubbles. The cell was placed into the ZetaSizer chamber and equilibrated at 25°C for 120 s. The suitable parameters chosen were, for material (colloidal gold): refractive index 0.18 and absorption 3.933, for dispersant (water): refractive index 1.33 and viscosity 0.8872 mPa/s at 25 ± 0.1°C. Quintuplicates of each sample were measured and each measurement comprised 20 runs to obtain a stable reading. Results were analyzed using the ZS Xplorer software.

#### 2.4.5 Transmission electron microscopy

The morphology of the AuNPs was examined with a highly integrated compact JEM-1010 transmission electron microscope (TEM; JEOL, Peabody, MA, United States) operated at an accelerating voltage of 30 kV. The transmission electron microscopy (TEM) is accessorized with a 2 k × 2 k AMT CCD camera for digital image acquisition. TEM grids were prepared by placing one 10 μL drop of a 3 μM AuNPs solution on carbon-coated copper grids which were dried at room temperature. The ImageJ 1.52 version software (U. S. National Institutes of Health) was used to determine the average diameter of AuNPs in the TEM images. A minimum of 200 particles were analyzed.

### 2.5 Detection of aflatoxin B_1_

#### 2.5.1 AFB_1_ stock solution

A primary standard solution (100 μg AFB_1_/mL) was prepared in dimethyl sulfoxide; subsequently, the solution was diluted to 1 μg AFB_1_/mL using Milli-Q water.

#### 2.5.2 Colorimetric assay

Different concentrations of AFB_1_ (3.125, 6.25, 12.5, 25, 50, 100, and 200 ng AFB_1_/mL) were introduced to a series of borosilicate glass tubes containing equal quantities of copper (II) chloride at a final concentration of 31.2 μM. Afterward, 500 μL of AuNPs-Lys (final concentration 0.3 mM) were added to each tube, the solution was vortexed and then equilibrated for 5 min at room temperature. The final volume of the system was 1 mL. The resultant UV–Vis absorption spectra were acquired in the range of 400–1,000 nm using a microplate spectrophotometer (Multiskan Sky, Thermo Scientific, Waltham, MA, United States). Furthermore, a calibration curve for AFB_1_ was plotted based on the ratio of absorbance at 625 and 522 nm (A_625_/A_522_). The limits of detection (LOD) and quantification (LOQ) were calculated as 3σ/m and 10σ/m, respectively. In the mathematical expressions, σ is the standard deviation of the blank (*n* = 10) and *m* is the slope of the calibration curve. Repeatability (relative standard deviation average, RSD%) was also calculated by 10 independent trials in the same laboratory by the same operator.

#### 2.5.3 Detection of AFB_1_ in maize samples and method validation

The performance of the colorimetric assay was tested by means of the 991.31 AOAC methodology (13), using antibody-based immunoaffinity columns for AFB_1_. The recovery of the toxin was evaluated in maize samples spiked with four replicates of six different AFB_1_ contents (6.25, 12.5, 25, 50, 100, and 200 ng AFB_1_/g). Before the contamination process, the commercial Supremo hybrid maize (Aspros Semillas, Cortazar, Guanajuato, Mexico) was analyzed for AFB_1_. Subsequently, the AFB_1_-contaminated maize samples were extracted by blending with methanol-water (80:20 v/v) and 5 g of non-iodized NaCl. The mixture was filtered through a Whatman 1 filter paper (solution A), and 5 mL were diluted with 20 mL of Milli-Q water (solution B). Solution B was filtered again through a micro-fiber filter, and 1 mL was passed through the immunoaffinity column (Afla B, VICAM Science Technology, Watertown, MA, United States). Subsequently, the column was washed with 10 mL of Milli-Q water and dried with sterile airflow. The toxin was then eluted with 1 mL of HPLC grade methanol and quantified in a fluorometer VICAM Series-4EX (VICAM Source Scientific. Irvine, CA, United States) after reaction with 1 mL of 0.002% aqueous bromine. The detection limit via fluorescence measurement is approximately 0.5 ng AFB_1_/g. The filtered solution B was also used for further detection of AFB_1_ via the colorimetric assay.

### 2.6 Experimental design and statistical analysis

The experiment was conducted as a completely randomized design. Data was assessed by analysis of variance (ANOVA) and means were separated according to the Tukey honest significant difference *post hoc* test using the Minitab 16.0.1 software (Penn State University, State College, PA, United States). The regression analysis was also performed with Minitab. In both cases, a significance value of *p* < 0.05 was considered to reject the null hypothesis.

## 3 Results and discussion

### 3.1 Gold nanoparticles: synthesis, functionalization, and characterization

#### 3.1.1 UV–Vis spectroscopy

**Figure 1:**
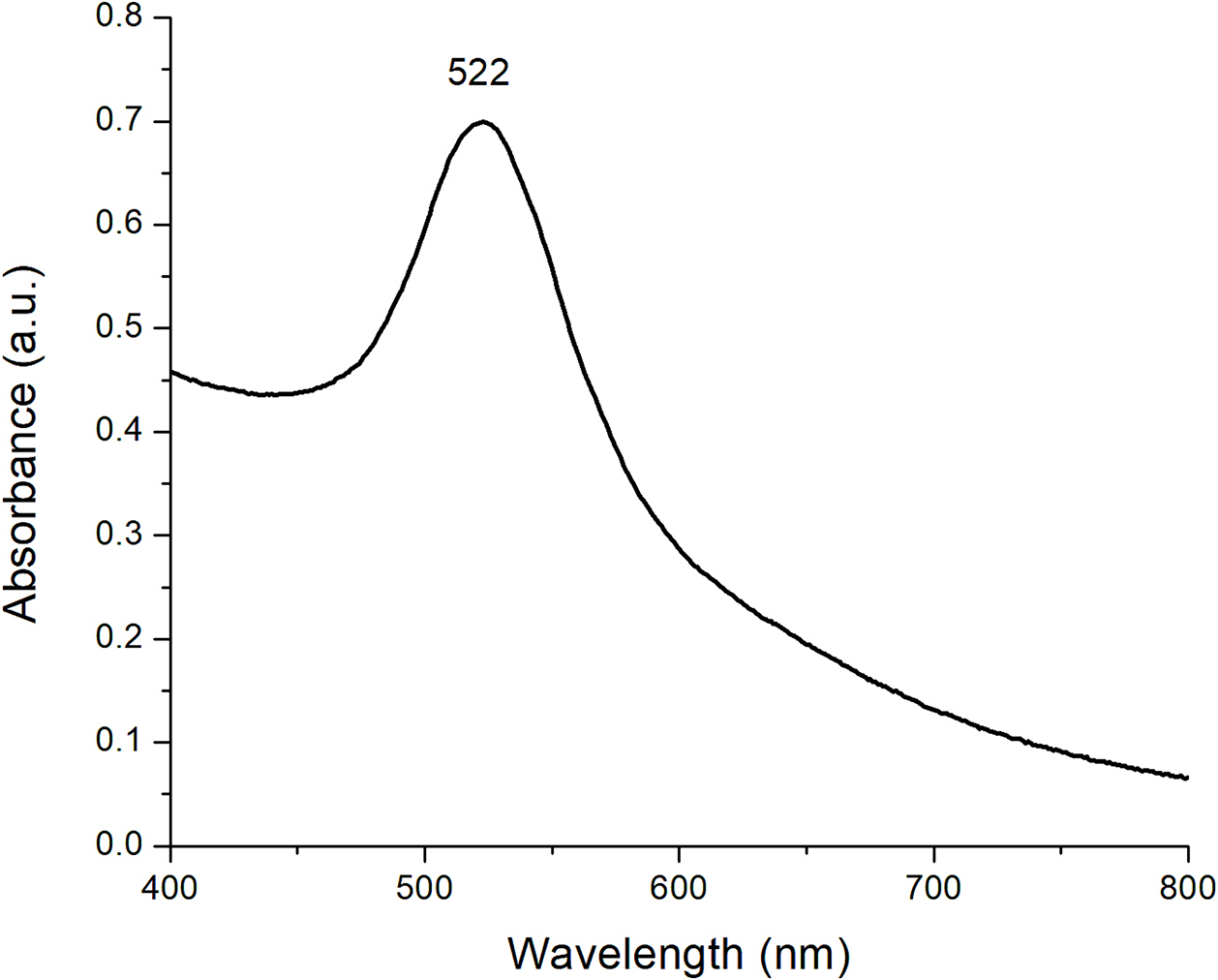
Representative UV–Vis absorption spectra of AuNPs (0.3 mM).

Figure 1 shows the UV–Vis absorption spectra of the as-prepared AuNPs at a concentration of 0.3 mM. As can be observed, there was a prominent absorption band centered at 522 nm due to the excitation of the SPR. The SPR was originated from light wave resonance with collective oscillation of conduction electrons at the AuNPs surface. Both the shape and position of the absorption band can be associated with 0-dimensional nanostructures with a spherical morphology (14). These results are in close agreement with the findings reported by Wang et al. (15) who stated that AuNPs synthesized using sodium citrate show a distinctive plasmon around 524 nm. Nanoparticle formation can also be tracked through the solution’s color shift, from colorless right after the addition of sodium citrate to a bright ruby-red shade when the reaction finishes.

**Figure 2:**
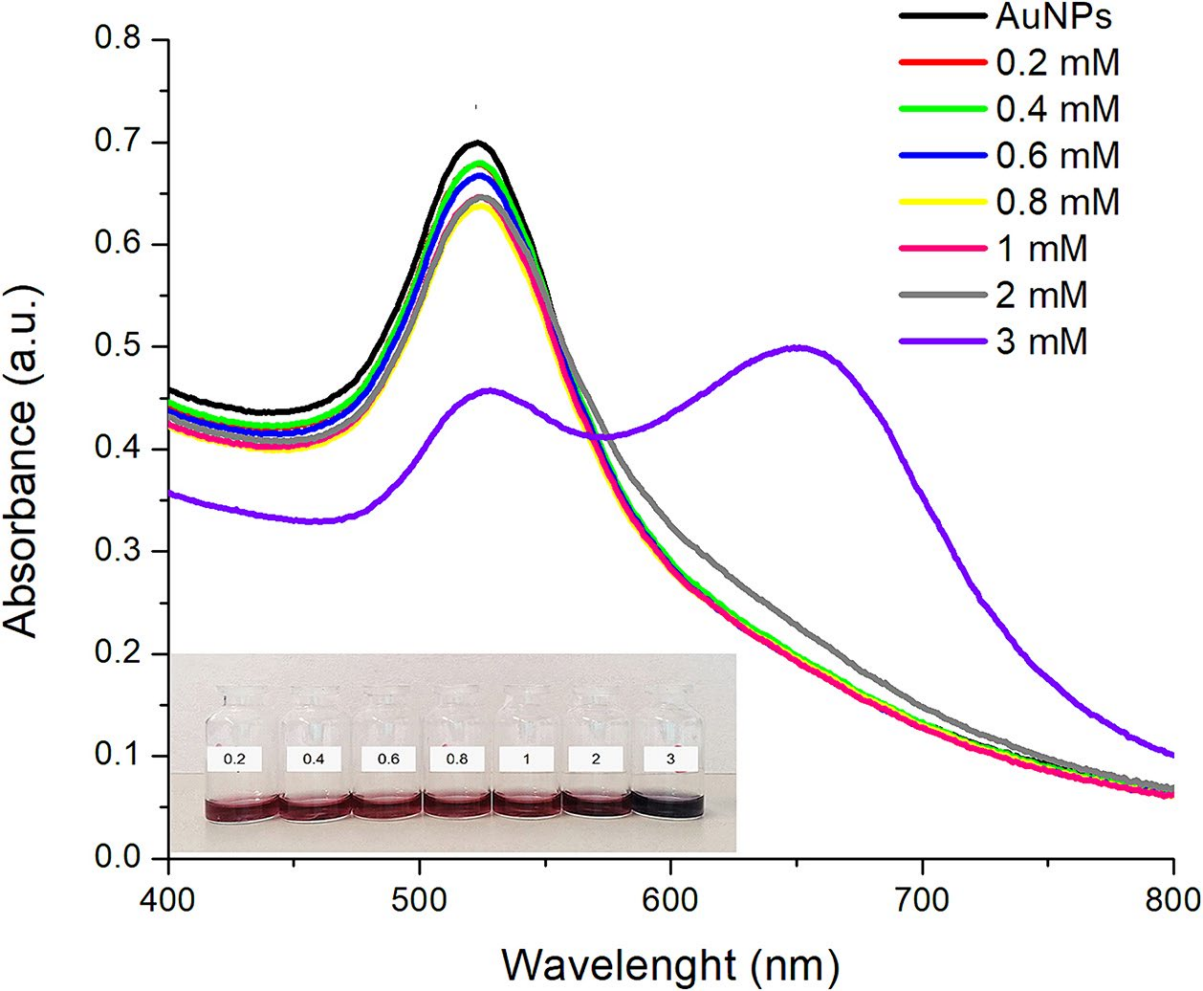
UV–Vis absorption spectra of the synthesized AuNPs (black line) and AuNPs functionalized with different concentrations of L-lysine at room temperature during 30 min. The inset shows a digital photograph of the AuNPs illustrating their corresponding colorimetric response.

Figure 2 shows the UV–Vis absorption spectra of AuNPs functionalized with different concentrations of L-lysine. It is observed that, when the L-lysine concentration increased up to 1 mM, the absorption maximum showed a slight red shift (higher wavelength) which became more notorious (~3 nm) with the increase in L-lysine concentration. Alongside this red shift, a decrease in the absorbance intensity was also observed. Moreover, when AuNPs were functionalized with the highest concentration of L-lysine (3 mM), a significant decrease in the absorbance intensity and an additional plasmon at around 650 nm were noticed (Figure 2).

In general, the decrease in plasmon intensity and the red shift are associated with an aggregation process, due to bonding between the positively charged amine groups of the L-lysine molecules surrounding the AuNPs’ surface and the negatively charged carboxyl groups of the sodium citrate used to stabilize the AuNPs (16, 17). It has been previously reported that at L-lysine concentrations ≤2.5 mM, AuNPs functionalization takes place through the formation of a “crown” of L-lysine molecules, without drastically altering the shape and position of the surface plasmon resonance (16). In this context, Horovitz et al. (16) reported that when changing the L-lysine concentration from 0.01 M to 0.54 M, a significant decrease in the absorbance accompanied with a red shift of the absorption maxima of AuNPs was observed. Moreover, it has been well described that, at higher concentrations, L-lysine molecules can also establish bonds among them, and subsequently self-assemble in a necklace-like structure leading to a shift in the SPR and the apparition of another plasmonic mode at larger wavelengths (17). In this research, this effect was confirmed, since AuNPs functionalized with the highest concentration of L-lysine (3 mM) showed a double plasmon due to the self-assembly via hydrogen bonding (Figure 2). The red shift was observed at AuNPs:L-lysine molar ratios greater than 1:2.7 suggesting that the AuNPs-Lys’ stability relies on this parameter. Moreover, a color shift is also associated with a self-assembling or an aggregation process; thus, for large self-assembled particles, the ruby color of the AuNPs-Lys system shifts to purple and then to a blueish-gray color (18). Furthermore, Figure 2 (inset), shows the AuNPs-Lys’ color, and no evident color change was observed for L-lysine concentrations between 0.2 and 1 mM. However, at a concentration of 2 mM, the suspension acquired a purple coloration, suggesting an increased assembly formation. The most evident change was observed at a concentration of 3 mM, where the coloration was blueish-gray. Along with the obtained UV–Vis spectra, these results suggest that AuNPs have gone through a self-assembly process to form aggregates. Since this work’s focus was the colorimetric detection of AFB_1_, it is important that the AuNPs do not show signs of aggregation during their functionalization with L-lysine and remain stable through time. With this consideration in mind, the AuNPs:L-lysine molar ratio was set at 1:2 for subsequent experiments.

#### 3.1.2 Fluorescence spectroscopy

**Figure 3:**
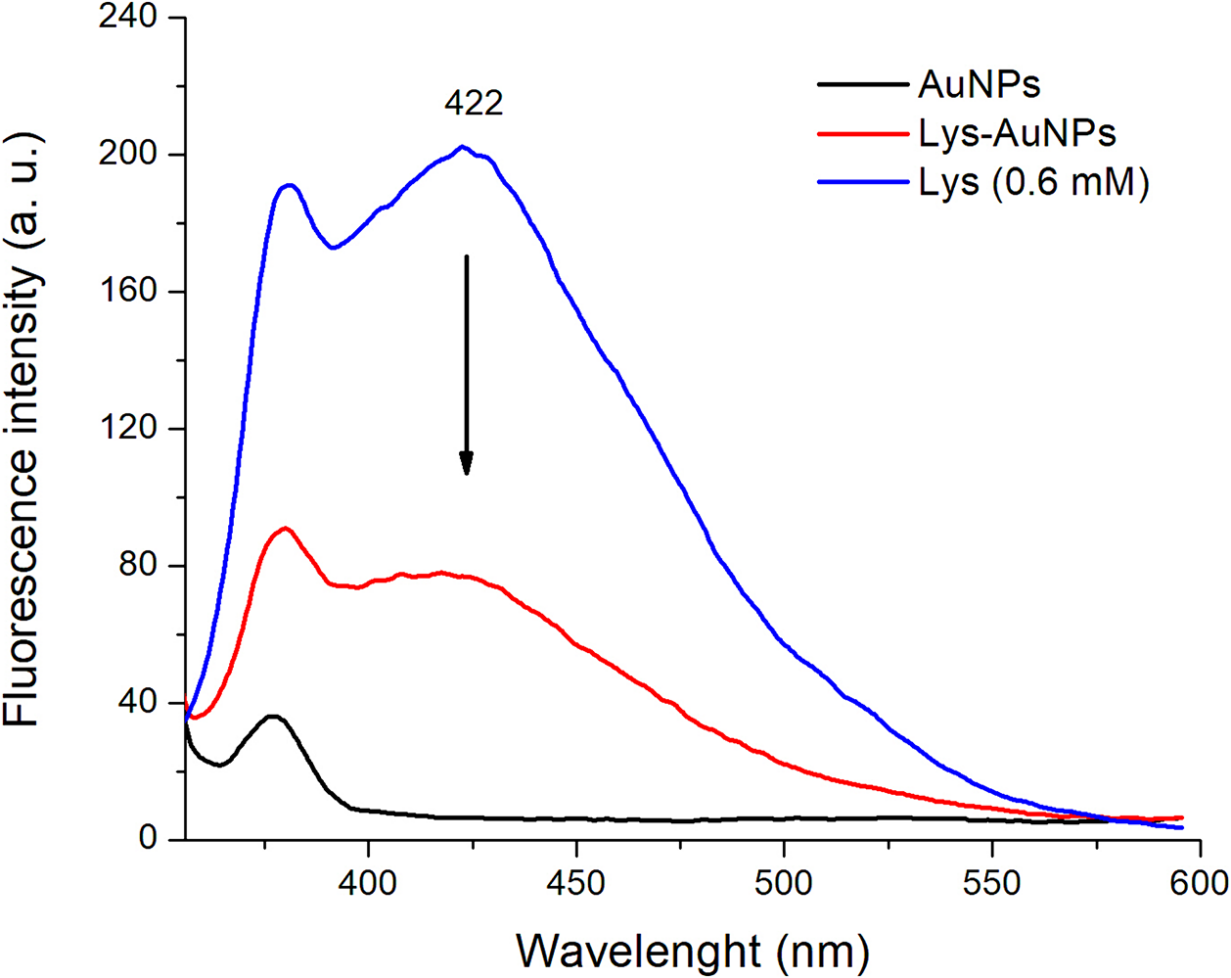
Fluorescence spectra of AuNPs, L-lysine, and AuNPs-Lys (molar ratio 1:2).

Figure 3 shows the fluorescence spectra of AuNPs (0.3 mM), L-lysine (0.6 mM), and AuNPs-Lys (functionalized with L-lysine at a concentration of 0.6 mM). The background-fluorescence emission (*λ*_ex_ = 330 nm) of AuNPs is presented to compare the spectrum of the AuNPs-Lys. In general, the L-lysine aqueous solution showed a considerable fluorescence band in the visible region with a maximum at 422 nm. It is well known that the fluorescence of L-lysine is correlated with the formation of aggregates and intermolecular interactions in the solution, as fluorescence does not originate merely from the structural properties of the amino acid. In this context, Stagi et al. (19) reported that an aqueous solution of L-lysine (0.1 M) showed a broad fluorescence band with a maximum at 415 nm. Moreover, AuNPs-Lys showed a moderate fluorescence band, confirming the successful functionalization of the AuNPs with the amino acid. Xu et al. (20) synthesized lysine-functionalized gold nanoclusters by using a lysine-mediating one-pot methodology. The authors reported that the aqueous solution of the as-prepared gold nanoclusters was highly fluorescent, showing a maximum emission peak at 418 nm (*λ*_ex_ = 338 nm). These results are consistent with our findings.

#### 3.1.3 FTIR spectroscopy

**Figure 4:**
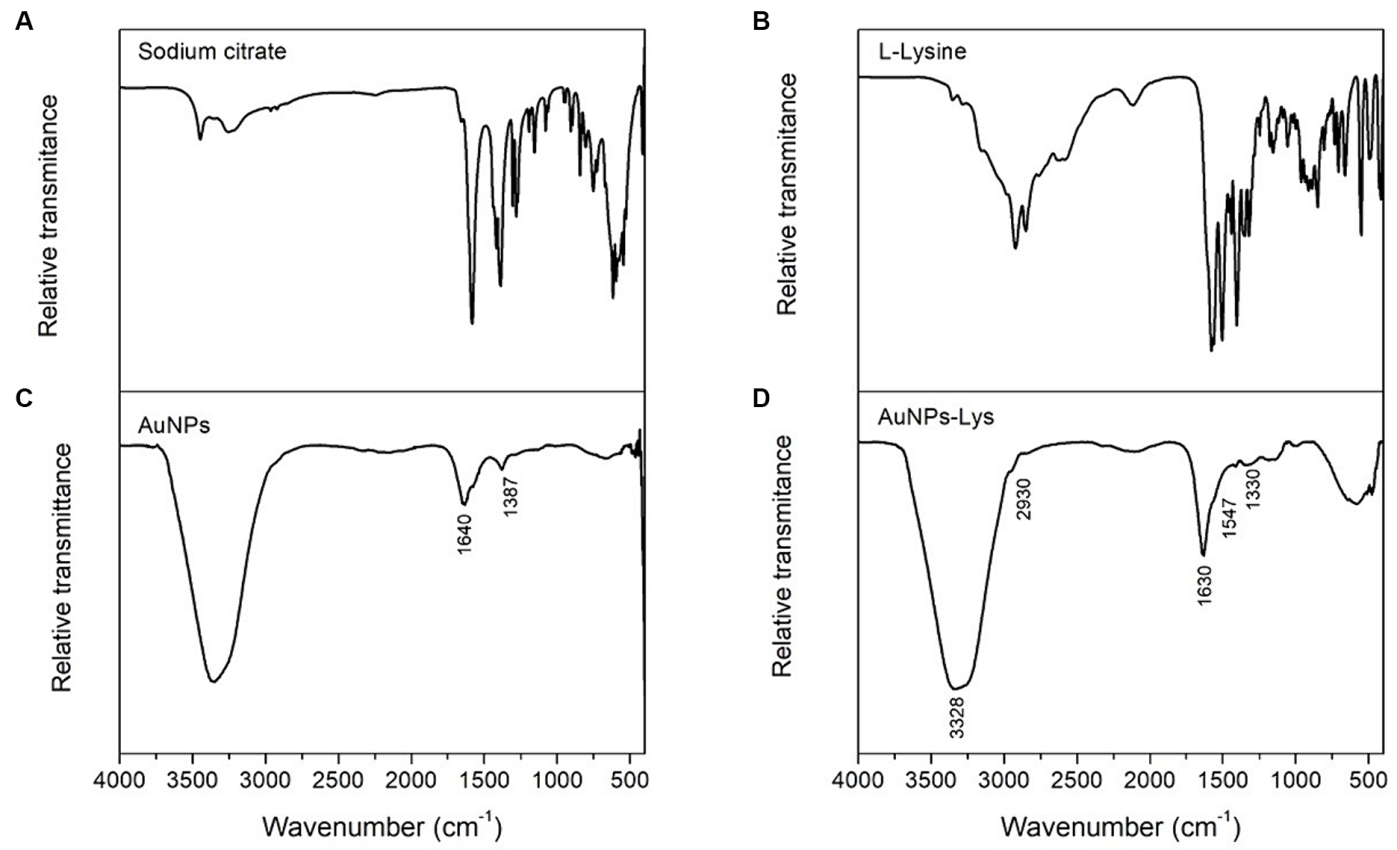
FTIR spectra of sodium citrate **(A)**, L-Lysine **(B)**, AuNPs **(C)**, and AuNPs-Lys molar ratio 1:2 **(D)**.

Figure 4 shows the FTIR spectra of sodium citrate, AuNPs, L-lysine, and AuNPs-Lys. In general, the citrate-capped AuNPs (Figure 4B) showed a considerable absorption band centered at 1,640 cm^−1^ associated with the C=O symmetric stretch, and another vibrational band at around 1,387 cm^−1^ assigned to the C–H stretching vibration. These findings, clearly confirm that sodium citrate moieties were attached to the surface of AuNPs. Moreover, AuNPs-Lys showed absorption bands corresponding to the amine and carboxyl groups present in the L-lysine molecule (Figure 4D). In concordance with the findings of Tsalsabila et al. (21) and Durmus et al. (22) it is possible to identify the bands at 3,328, 1,547, and 1,330 cm^−1^ corresponding to the N–H vibration. Moreover, the band at 2,930 cm^−1^ is associated with the C–H vibration, and the band around 1,630 cm^−1^ is related to the C=O stretching vibration of the amino acid (Figure 4C). It is well known that the self-assembly of metal nanoparticles with amino acids occurs via the formation of hydrogen bonding between carboxyl and amine groups. Thus, as shown in Figure 4D, the band intensity in the 3,328–1,630 cm^−1^ region corresponding to the N–H stretching (amide A group) and C=O stretching (amide I group) of the AuNPs-Lys became stronger confirming their successful functionalization with the amino acid.

#### 3.1.4 Size distribution by NTA and DLS, and zeta potential

**Figure 5:**
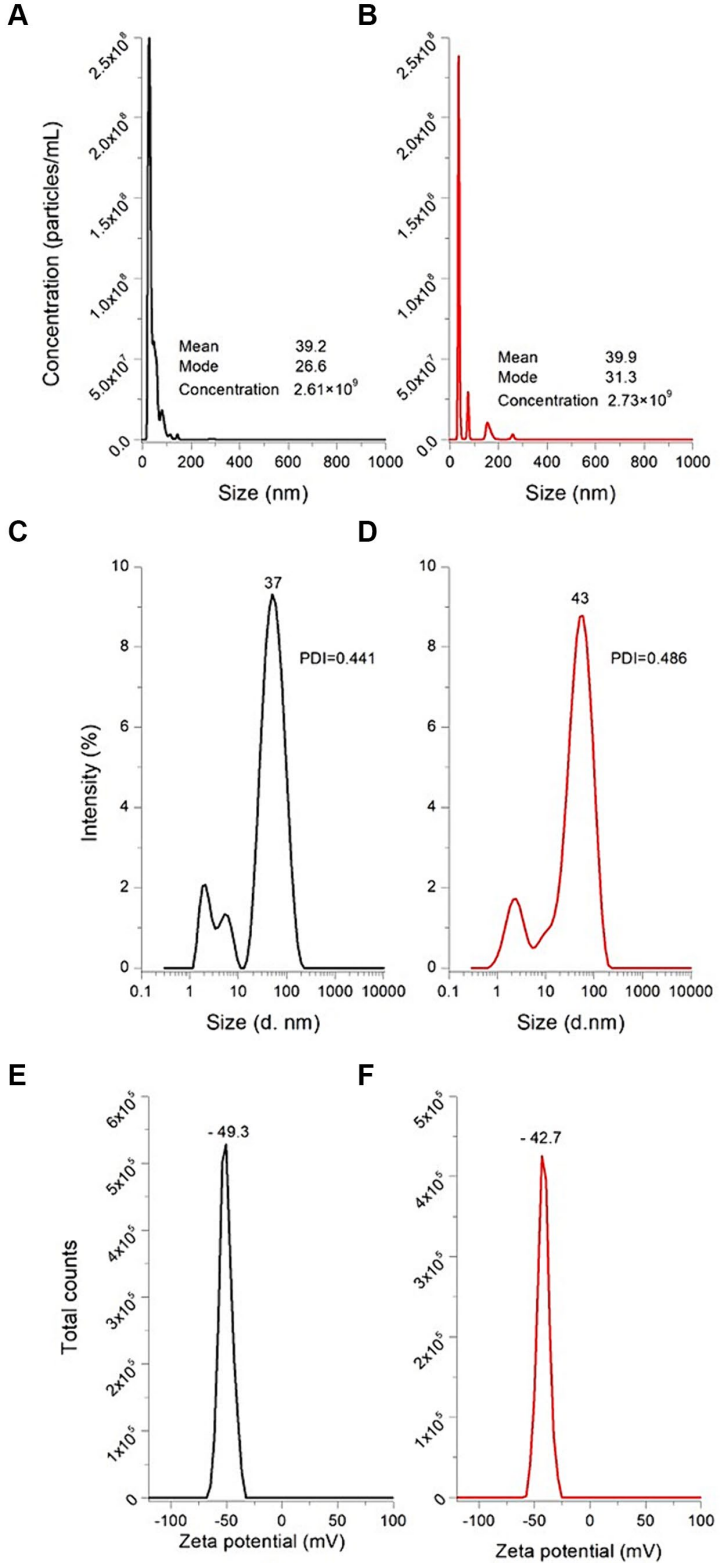
Particle size distribution and concentration of AuNPs **(A)** and AuNPs-Lys **(B)** measured by NTA, particle size distribution of AuNPs **(C)** and AuNPs-Lys **(D)** measured by the DLS technique, and zeta potential of AuNPs **(E)** and AuNPs-Lys **(F)** measured by Doppler electrophoresis. PDI, polydispersity index.

The particle size distribution of the as-prepared AuNPs, confirmed using the NTA technique, is shown in Figure 5A. In general, there was a nonparametric distribution of the AuNPs, with the majority ranging around 26.6 nm in size, and two other minor populations with bigger diameters (41 nm and 68 nm, respectively). The mean value calculated from the NTA software was 39.2 nm. Regarding particle concentration, the AuNPs diluted preparation dropped 2.61 × 10^9^ particles per mL. Consequently, each experimental unit at a concentration of 0.3 mM contained approximately 2.61 × 10^11^ particles/mL. Malik et al. (23) synthesized AuNPs from chloroauric acid (0.225 mM) through Turkevich’s methodology, and the AuNPs were characterized utilizing the NTA technique. The authors reported that the average size of the as-prepared AuNPs was 35 nm. This result is similar and consistent to those shown in this work; however, the variation in size may be due to the differences in the gold:citrate molar ratio, since it is well known that this ratio is one of the parameters that modulates nanoparticle size. Moreover, Figure 5B shows the NTA results obtained for the AuNPs-Lys (molar ratio 1:2). In general, when AuNPs were functionalized with L-lysine, the mean size did not significantly change (mean value 39.9 nm and mode value 31.3 nm). Due to the interaction between AuNPs and L-lysine molecules, there was a slight increase in size, because of the formation of hydrogen bonding between the –NH_2_ and –COOH groups (16). These results are consistent with those observed during the optical characterization. Furthermore, from the NTA results, it was possible to observe that the concentration of the AuNPs-Lys diluted preparation was the same as those recorded for AuNPs, confirming that the number of particles per mL in the stock did not differ statistically (2.73 × 10^11^).

Figure 5 also depicts the intensity distributions of AuNPs and AuNPs-Lys (molar ratio 1:2) measured by the DLS technique. In general, the as-prepared AuNPs are monodisperse [polydispersity index (PDI) 0.441] with an average hydrodynamic diameter of about 37 nm (Figure 5C). However, when L-lysine (0.6 mM) was incubated with the AuNPs for 30 min at room temperature, the hydrodynamic diameter of the particles increased slightly, up to 43 nm (Figure 5C) yielding a polydispersity index of 0.486. This fact confirms the successful functionalization of the metal nanoparticles; therefore, L-lysine was found to be appropriately assembled on the AuNPs surface. In general, the obtained size values with the DLS technique slightly differ from those attained with the NTA methodology. In this context, James et al. (24) reported that DLS has disadvantages in identifying individual particles with different sizes in polydisperse samples, more still when the particle sizes are relatively small. Thus, the DLS technique shows higher sensitivity for bigger particles. According to the data presented by Hou et al. (25) this phenomenon is because DLS evaluates and compares the intensity of light scattered due to particle movement, whereas NTA is based on the tracking of individual particles and the analysis of their velocity (Brownian movement). Consequently, DLS has been proved to have a lower detection limit for small particles when compared to NTA.

On the other hand, zeta potential is an indicative parameter of nanoparticle stability in the suspension, and nanoparticle’s surface charge. For electrostatic stabilization, values above ±30 mV are required (26). In general, AuNPs exhibited a zeta potential value of −49.3 mV (Figure 5E), indicating their excellent stability with a highly electronegative surface charge. These results are in close agreement with those obtained by Wang et al. (27) who reported a zeta potential value of around −40 mV for AuNPs synthesized through Turkevich’s method. In contrast, AuNPs-Lys experienced a slight change in their zeta potential reaching values up to −42.7 mV (Figure 5F), confirming the modification of the nanoparticle’s surface charge upon functionalization. It has been reported that the binding of L-lysine with the AuNPs surface could occur through electrostatic interactions between the lysine’s protonated ε-amine group and the negatively charged citrate anions adsorbed onto the gold surface (16). In this research, the zeta potential absolute value was greater than 30 mV, indicative that the AuNPs-Lys will remain stable over time (the AuNPs-Lys were stable in the original solution for over 100 days).

#### 3.1.5 TEM

**Figure 6:**
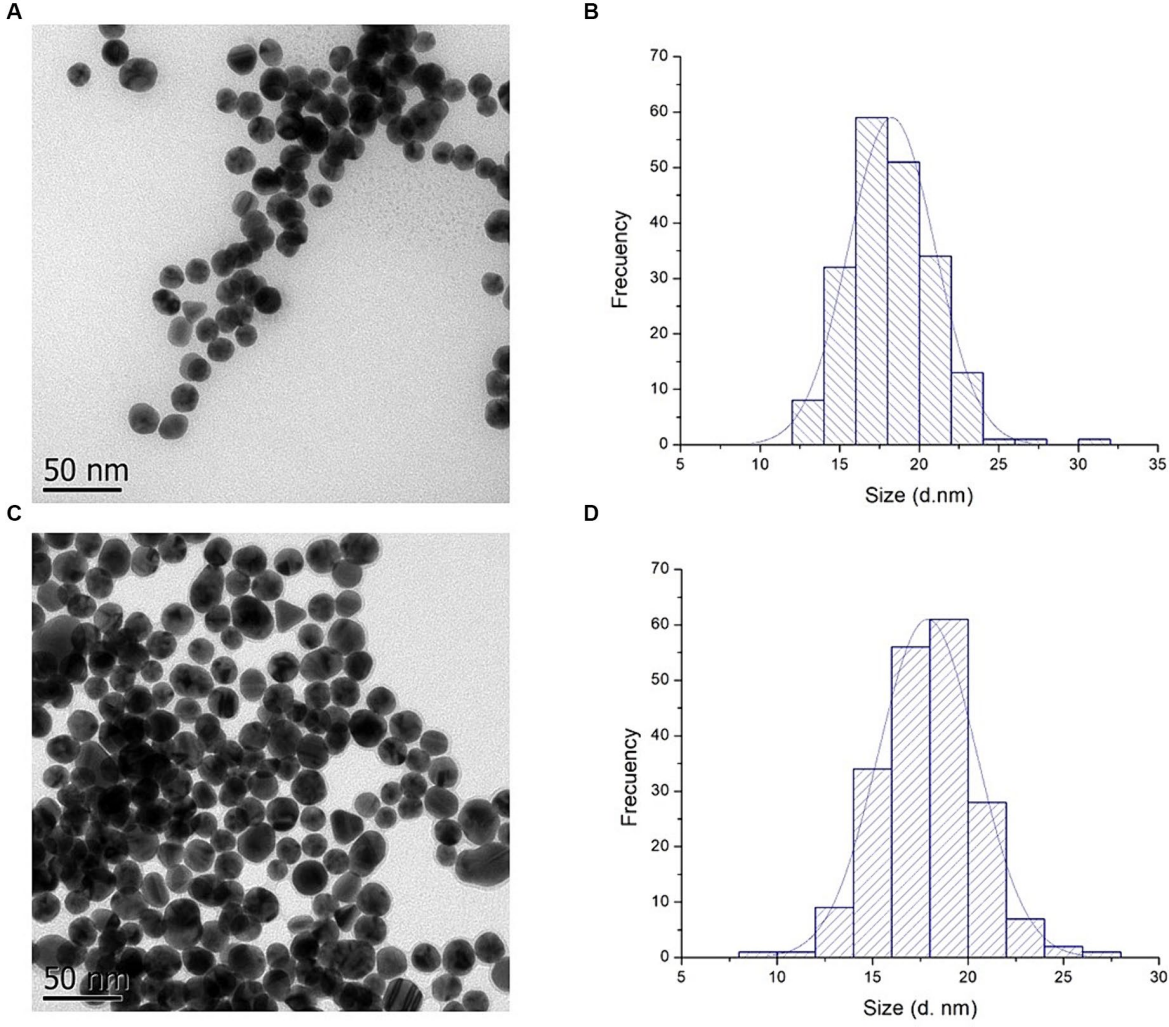
TEM images **(A,C)** and particle size histogram **(B,D)** of AuNPs and AuNPs-Lys at molar ratio 1:2, respectively.

To evaluate the morphology and confirm the size of AuNPs and AuNPs-Lys, transmission electron microscopy was used. As shown in Figure 6, the majority of the AuNPs were quasi-spherical in shape. Moreover, a quantitative image analysis of the TEM micrographs revealed average sizes of 17.87 ± 2.56 and 18.27 ± 2.71 nm for AuNPs and AuNPs-Lys, respectively (Figures 6A,C). The corresponding size distribution histogram is shown in Figures 6B,D. The average diameter of the AuNPs and AuNPs-Lys slightly differ from those obtained by the NTA and DLS techniques. This phenomenon could be due that the hydrodynamic diameter considers surface-bound ions and molecules as well as the layer of hydration around the nanoparticle surface. Moreover, TEM characterization confirmed the results obtained by UV–Vis and DLS, demonstrating that AuNPs and AuNPs-Lys were monodispersed without any detectable aggregation.

### 3.2 Colorimetric assay

#### 3.2.1 The interaction of AuNPs-Lys with Cu^2+^ ions

**Figure 7:**
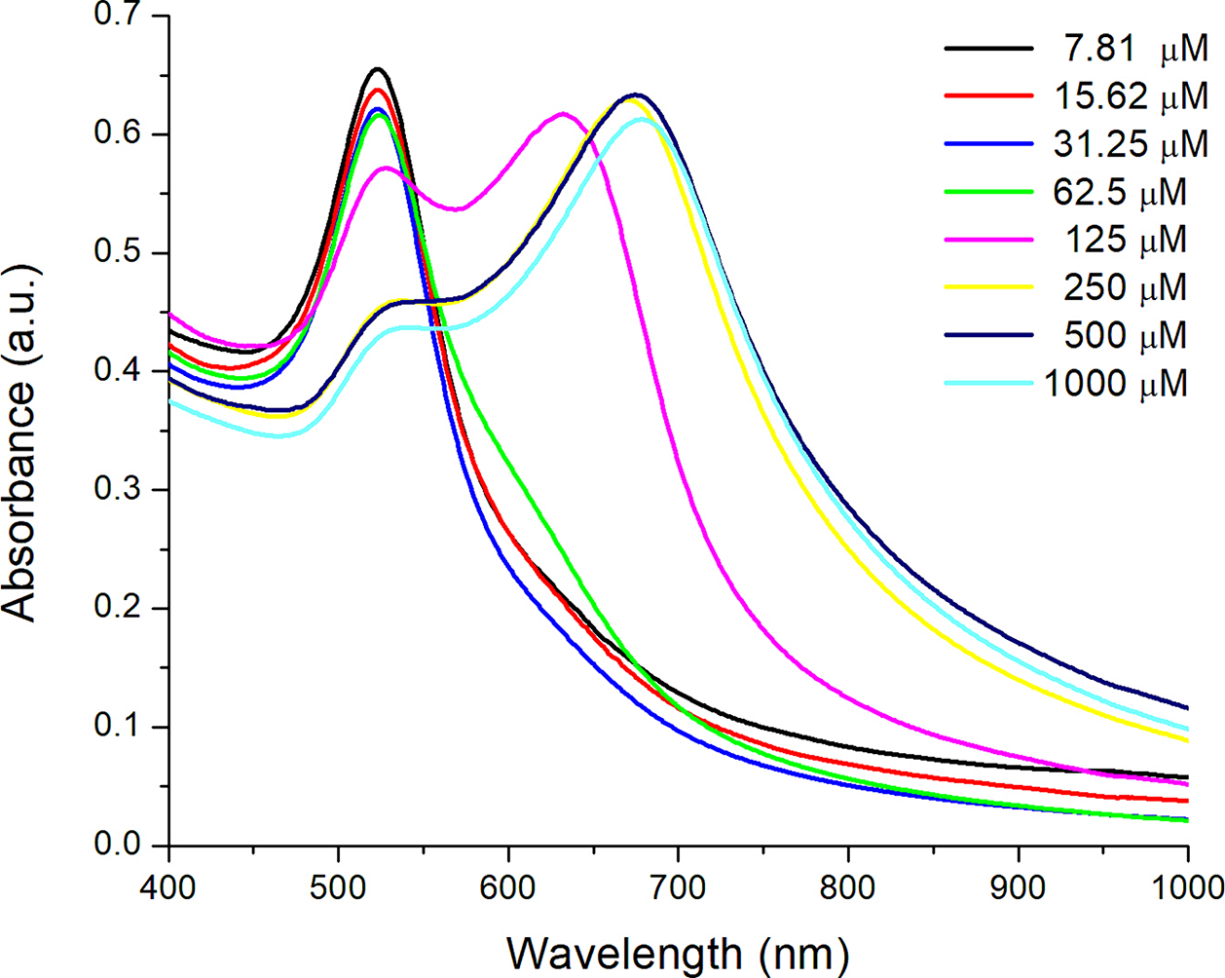
UV–Vis absorption spectra of AuNPs-Lys (molar ratio 1:2) in the presence of different concentrations of copper (II) chloride.

Figure 7 shows the UV–Vis absorption spectra of AuNPs-Lys (molar ratio 1:2) in the presence of different concentrations of copper (II) chloride (from 7.81 to 1,000 μM). In general, as the copper concentration increased, the SPR corresponding to the AuNPs (522 nm) significantly decreased in intensity and shifted to higher wavelengths (red shift). However, when the copper concentration was above 62.5 μM, a small shoulder appeared (Figure 7), related to nanoparticle aggregation (an increasing assembly formation of AuNPs). From a copper concentration of 125 μM onwards, the plasmon shape changed drastically, with the appearance of a second absorption maximum at around 680 nm, indicating the formation of large self-assembled structures (20). Thus, it is observed that L-lysine favored the aggregation of AuNPs in the presence of an excess of Cu^2+^ ions in a short period. In this regard, L-lysine contains two amino groups, one for the interaction with the negatively charged citrate adsorbed on the gold surface and the other available for the formation of copper chelates. In this work, it was observed that copper concentrations between 7.81 and 31.25 μM were enough to avoid nanoparticle aggregation (Figure 7). Consequently, the concentration of copper chloride needed to get an adequate optical response in the presence of AFB_1_ molecules was set at 31.2 μM. In this context, Sener et al. (28) synthesized AuNPs-Lys for the detection of Hg^2+^ in aqueous media. The authors reported that at high Hg^2+^ concentrations, a second plasmon appeared at around 725 nm, representative of the self-assembly of the AuNPs because of their interaction with Hg^2+^ ions. This data supports the findings obtained in this research.

#### 3.2.2 AFB_1_ detection

**Figure 8:**
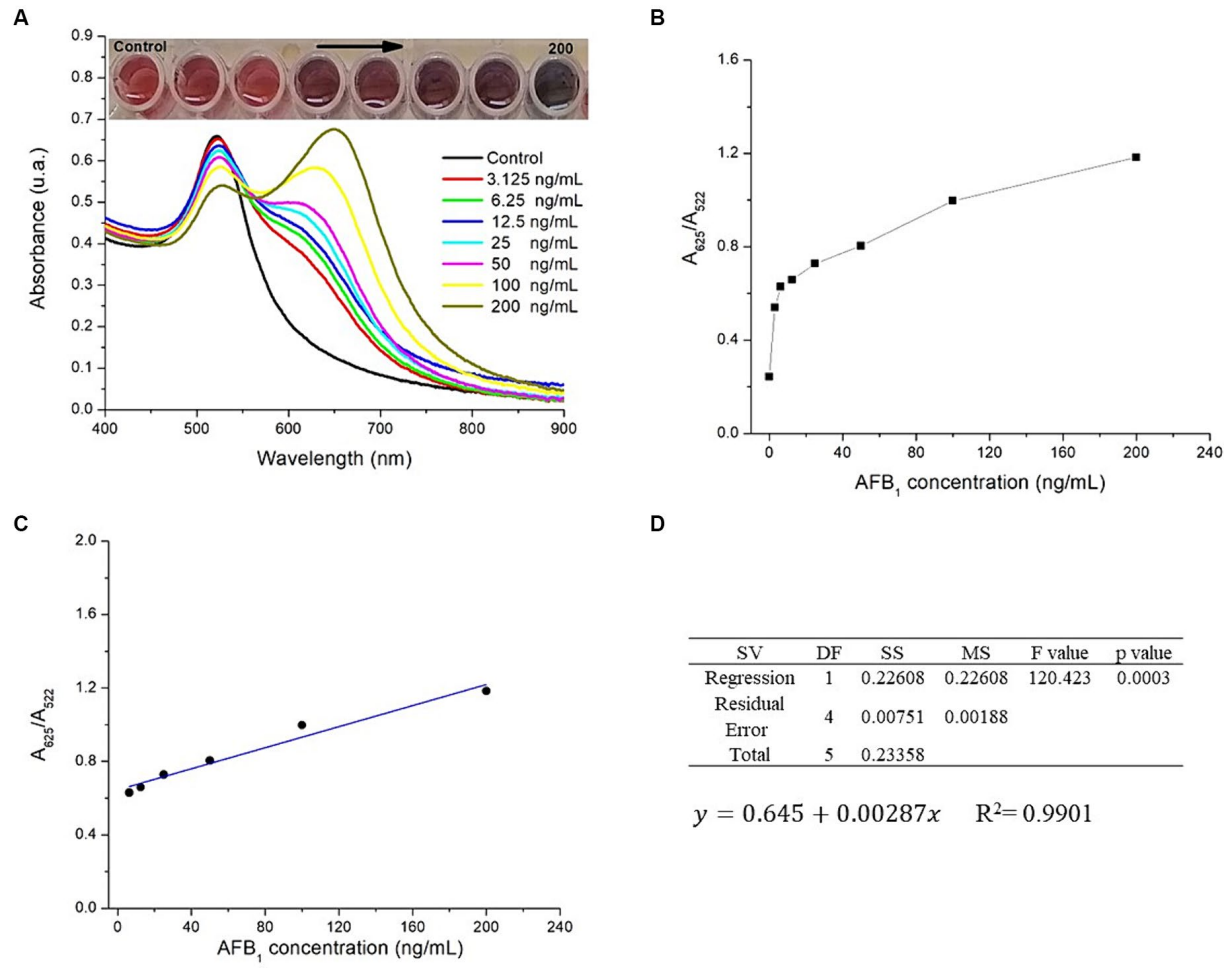
**(A)** UV–Vis absorption spectra of the AuNPs-Lys system exposed to different concentrations of AFB_1_ (ng/mL) in the presence of copper ions, **(B)** A_625_/A_522_ ratio versus the concentration of AFB_1_, **(C)** linear correlation over the concentration range of 6.25 to 200 ng AFB_1_/mL, **(D)** the ANOVA table at an *α*-level of 0.05 and the model regression equation.

AFB_1_ determination using AuNPs-Lys was based on their aggregation. This aggregation phenomenon produced a color change and a notorious variation in the absorbance signal. Figure 8A shows the UV–Vis absorption spectra of the AuNPs-Lys system after interacting with a range of concentrations of AFB_1_ in the presence of Cu^2+^ ions. Overall, there was a significant formation of a secondary plasmon at higher wavelengths (~ 650 nm) when the mycotoxin concentration increased from 3.125 to 200 ng AFB_1_/mL. While at concentrations below 50 ng AFB_1_/mL the secondary plasmon’s absorption was lower than that of the main AuNPs plasmon, this behavior was reversed at concentrations above 50 ng AFB_1_/mL, which suggests that AFB_1_ molecules greatly enhance the formation of aggregates with the subsequent change in the nanoparticle’s arrangement in the form of networks. This phenomenon was also accompanied by a significant color change in the system, from wine red to purple to a bluish tone. According to the obtained data, the maximum degree of self-assembly of the AuNPs-Lys became more evident above 100 ng AFB_1_/mL, suggesting that at higher AFB_1_ concentrations the self-assembly will be greater.

The aggregation effect of the AuNPs-Lys system when interacting with AFB_1_ molecules in the presence of Cu^2+^ ions was further evaluated by the absorbance ratio (A_625_/A_522_) as plotted in Figure 8B. As can be observed, the A_625_/A_522_ value increased gradually over the AFB_1_ concentration. However, a linear correlation existed over the mycotoxin concentration range of 6.25 to 200 ng AFB_1_/mL (Figure 8C). The linearity estimated with the coefficient of determination (*R*^2^) within the selected AFB_1_ range was 0.9901. Furthermore, the *p*-value in the ANOVA table, confirmed that the positive relationship between the AFB_1_ concentration and the A_625_/A_522_ ratio was statistically significant at an *α*-level of 0.05 (Figure 8D). The model regression equation to predict outcomes is also presented in Figure 8D. The LOD of the colorimetric assay was calculated to be 4.18 ng AFB_1_/mL, which is within the action level (20 ng/g) for total aflatoxins in maize intended for human consumption defined by the Mexican regulation NOM-188-SSA1-2002. The LOQ was 13.94 ng AFB_1_/mL. Repeatability was also calculated, the RSD average for the different AFB_1_ concentrations evaluated was 3.9%. In line with these results, Du et al. (11) designed a colorimetric method for the detection of total aflatoxins (AFs) based on the de-aggregation of AuNPs-Lys in the presence of Hg^2+^ ions. The authors reported a linear correlation in the range of 1.5–30 ng AFs/mL with a LOD of 1.1 ng AFs/mL. As can be seen, the LOD is slightly lower than the value obtained here (1.1 vs. 4.18 ng AFB_1_/mL); however, the methodology proposed in this research offers the possibility to quantify the contaminant in concentrations up to 200 ng AFB_1_/mL. Besides, the technique based on the aggregation of nanoparticles in the presence of copper ions can be an attractive option from the viewpoint of the minimization of toxic and hazardous contaminants such as mercury.

#### 3.2.3 Proposed mechanism of the colorimetric assay

**Figure 9:**
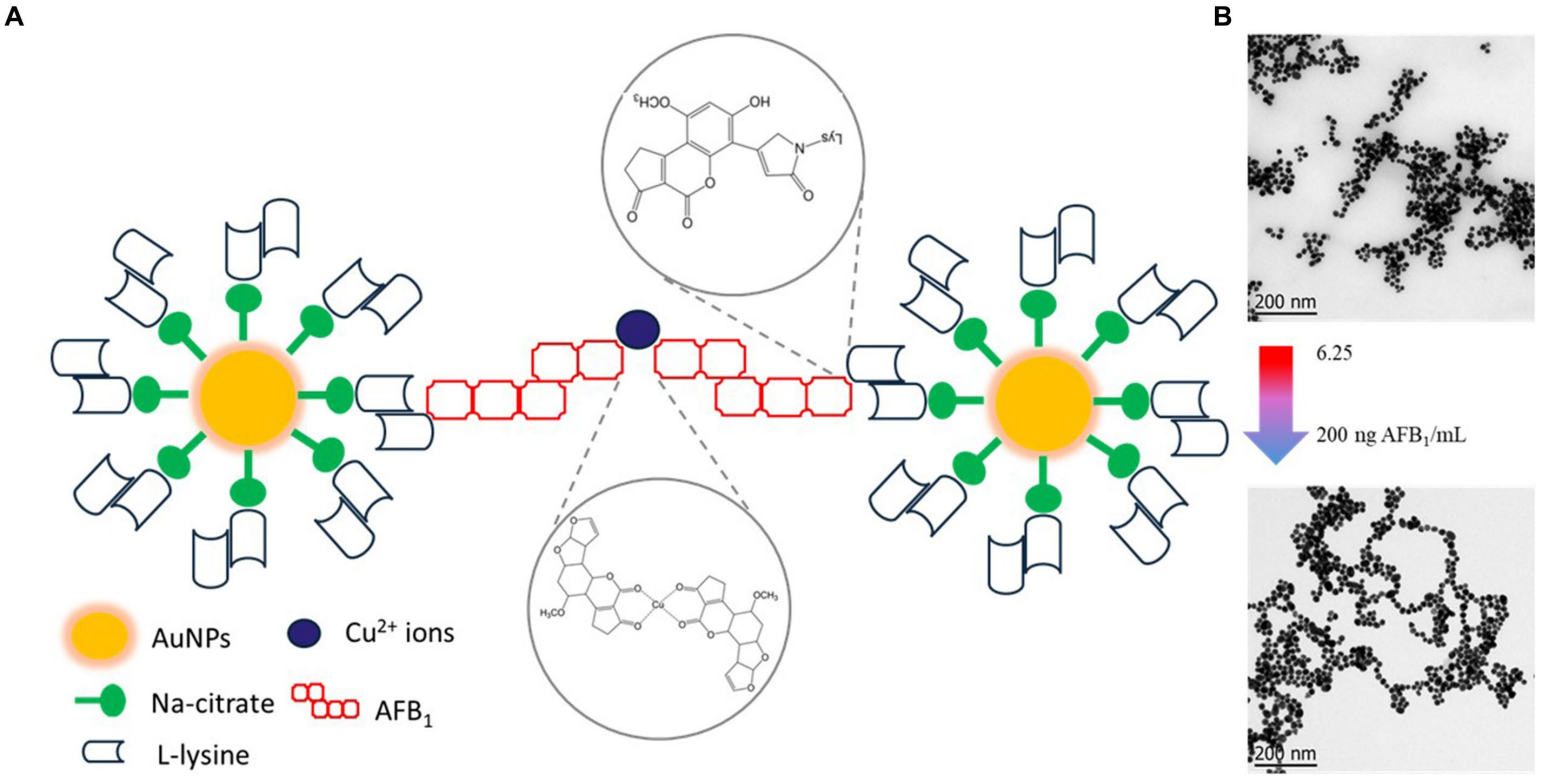
Schematic representation of the proposed mechanism for the colorimetric detection for AFB1 based on the aggregation of gold nanoparticles **(A)**, the formation of aggregates with the subsequent change in the nanoparticle’s arrangement **(B)**.

Figure 9A shows the proposed mechanism for the colorimetric detection of AFB_1_ based on the aggregation of nanoparticles. At the beginning of the reaction, copper ions interacted with the oxygen atoms of the β-dicarbonyl moiety of AFB_1_ molecules via cation attraction, forming AFB_1_-Cu (II) complexes. Subsequently, one free ε-amine group of the red dispersed AuNPs-Lys covalent binds to the AFB_1_-diol, produced through the oxidation of AFB_1_ molecules in the aqueous environment. Thus, as the concentration of AFB_1_ increased in the system, the formation of aggregates with the subsequent change in the nanoparticle’s arrangement in the form of large networks, was observed (Figure 9B). The formation of these networks was also accompanied by a drastic change in the color of the detection system, from wine red to purple to blueish gray. Thus, when the concentration of the mycotoxin exceeded 25 ng AFB_1_/mL, the red wine color promptly turned to blueish gray. This visual strategy could be very suitable for on-site rapid detection of AFB_1_ without any instrumentation because the operator can easily judge whether the mycotoxin is over the maximum permitted limit established by competent authorities by using the naked eye. However, the proposed mechanism of the colorimetric assay should be considered as a preliminary approach, considering that the matter is worthy of an in-depth study.

### 3.3 Detection of AFB_1_ in artificially contaminated maize samples and method validation

To evaluate the performance of the colorimetric assay in a real-world scenario, samples of maize were artificially contaminated with different contents of AFB_1,_ and the results were validated against an official AOAC methodology using monoclonal antibody immunoaffinity columns for AFB_1_. Data on performance parameters are summarized in Table 1. In general, there was a good agreement between the spiked AFB_1_ content and the measured values in both assays. The average recoveries for the colorimetric and immunoaffinity assays were between 91.2–98.4% and 96.0–99.2%, respectively. Furthermore, the relative standard deviations were satisfactory, showing values up to 7.7% and up to 5.0% for the colorimetric and immunoaffinity assays, respectively. These results indicated that the proposed methodology could be applicable to the determination of AFB_1_ in maize samples.

**Table 1:** Performance parameters of the colorimetric assay and the official methodology for the determination of AFB_1_ in maize samples.

AFB_1_ content ng/g	AuNPs-Lys system			991.31 AOAC methodology		
	Mean ± SD	Recovery	RSD	Mean ± SD	Recovery	RSD
6.25	5.8 ± 0.3	92.8	5.1	6.0 ± 0.3	96.0	5.0
12.5	11.4 ± 0.9	91.2	7.7	12.2 ± 0.5	97.6	4.1
25	23.1 ± 1.5	92.4	6.5	24.0 ± 0.9	96.0	3.8
50	47.9 ± 2.0	95.8	4.2	49.6 ± 1.3	99.2	2.7
100	98.4 ± 3.6	98.4	3.6	98.9 ± 1.8	98.9	1.8
200	194.3 ± 4.7	97.2	2.4	197.4 ± 2.0	98.7	1.1

Mean values ± standard deviation (*n* = 4). SD, standard deviation; RDS, relative standard deviation.

## 4 Conclusion

The as-prepared AuNPS-Lys (at a concentration of ~2.73 × 10^11^ particles) offered a clear colorimetric response in the presence of AFB_1_ and Cu^2+^ ions showing linearity in the range of 6.25–200 ng AFB_1_/mL, with a detection limit of 4.18 ng AFB_1_/mL. The reported can sense AFB_1_ in a wide range of concentrations via photometric or visual inspection. Moreover, the colorimetric assay can also be used as an eco-friendly and cost-effective methodology for the detection and quantification of AFB_1_ in maize samples. However, further research is needed to evaluate the anti-interference ability of the colorimetric technique when other structurally related mycotoxins such as sterigmatocystin—a precursor of AFB_1_—may be present in food or feedstuff matrixes. Research in this direction is in progress in our laboratories.

## Data availability statement

The original contributions presented in the study are included in the article/supplementary material, further inquiries can be directed to the corresponding author.

## Author contributions

KS-C: Data curation, Formal analysis, Investigation, Methodology, Software, Writing – original draft. DQ-G: Formal analysis, Supervision, Validation, Visualization, Writing – review & editing. MJ-Y: Formal analysis, Supervision, Validation, Visualization, Writing – review & editing. AM-A: Conceptualization, Formal analysis, Funding acquisition, Project administration, Resources, Visualization, Writing – review & editing. AV-D: Conceptualization, Formal analysis, Funding acquisition, Project administration, Resources, Visualization, Writing – review & editing.
